# Chitosan-Based Aerogel Cushioning Packaging for Improving Postharvest Quality of Wax Apples

**DOI:** 10.3390/foods15020192

**Published:** 2026-01-06

**Authors:** Yujie Hou, Sitong Zhou, Shiqi Liu, Peng Jin, Yonghua Zheng, Zhengguo Wu

**Affiliations:** College of Food Science and Technology, Nanjing Agricultural University, Nanjing 210095, China; m13579262311@163.com (Y.H.); 9221810622@stu.njau.edu.cn (S.Z.); lsss7@stu.njau.ed.cn (S.L.); pjin@njau.edu.cn (P.J.); zhengyh@njau.edu.cn (Y.Z.)

**Keywords:** polyvinyl alcohol, montmorillonite, biocompatibility, simulated transport vibration

## Abstract

Mechanical damage and microbial contamination are major challenges in the postharvest logistics of perishable fruit. In this study, two types of functionally modified chitosan-based aerogel pads were developed to enhance cushioning and preservation of wax apples. A chitosan/polyvinyl alcohol (CP) aerogel was first optimized by adjusting solid content, CS:PVA ratio, and crosslinker concentration. The optimal formulation (2% solids, 1:1 CS: PVA, 3% glutaraldehyde) exhibited a uniform porous structure and improved compressive strength. A chitosan/montmorillonite (CM) aerogel with 5% montmorillonite (MMT) showed high porosity, low density, and excellent cyclic stability. Incorporating 10% copper nanoparticle-loaded antibacterial fibers (CuNPs-TNF) into CM aerogels yielded CM-Cu aerogels with enhanced cushioning and antimicrobial properties. Under simulated transport and cold storage conditions, all aerogel-packaged groups reduced mechanical damage and decay of wax apples. Compared to the control, the CM-Cu group showed 66% lower decay, 5% less weight loss, 6 N greater firmness, 7% less juice yield, and a 13% reduction in relative electrical conductivity. Additionally, it better preserved fruit color and total soluble solids, extending shelf life by 4 d at 20 °C. These results demonstrate the potential of chitosan-based aerogels as multifunctional packaging materials that combine mechanical protection with antimicrobial activity for perishable fruit preservation.

## 1. Introduction

Wax apple (*Syzygium samarangense*) is a non-climacteric fruit cultivated in South China and Southeast Asian countries [1]. It is highly favored for its sweet and juicy taste, as well as its abundance of bioactive compounds such as polysaccharides [2], minerals [3], organic acids [4], and flavonoids [5], which confer both nutritional and medicinal benefits. However, the vigorous post-harvest respiratory metabolism of wax apples makes them extremely challenging to store. At room temperature, their shelf-life is merely 3 to 5 d, resulting in severe post-harvest losses. During transportation, the inevitable vibrations can cause the fruit to collide and be squeezed, leading to surface damage [6]. This not only directly reduces their commercial value but also makes them more susceptible to microbial infections [7], accelerating spoilage [8], causing nutrient loss [9], and posing risks to food safety [8]. Therefore, developing packaging materials with both cushioning and anti-microbial preservation functions is a crucial step in addressing the post-harvest losses of wax apples. Traditional cushioning materials, such as non-degradable foams and sponges, provide effective physical protection but lack additional functional properties and raise significant environmental concerns [10]. In contrast, ideal cushioning packaging for fresh-keeping applications should not only ensure excellent vibration damping and energy absorption but also possess antimicrobial properties and demonstrate environmental sustainability.

Chitosan (CS) has emerged as an ideal biomass material for aerogel fabrication due to its high structural tunability, abundant reactive functional groups, and excellent biocompatibility [11]. However, single-component chitosan aerogels suffer from limitations such as insufficient mechanical strength, weak antibacterial activity, and limited antioxidant capacity, which restrict their application in food packaging, especially for fresh produce preservation. To address the mechanical shortcomings, poly (vinyl alcohol) (PVA) is frequently incorporated. PVA is a water-soluble, non-toxic, and biodegradable synthetic polymer. Its molecular chains contain numerous hydroxyl groups, which can form hydrogen bonds with the amino groups on CS molecules [12]. This interaction facilitates thorough interpenetration of the molecular chains, helping to construct a robust intermolecular network and thereby enhance the aerogel’s mechanical properties [13]. Furthermore, chemical crosslinking is a key strategy for improving structural stability. Glutaraldehyde (GA) is a commonly used difunctional crosslinker whose aldehyde groups can react with hydroxyl groups, forming acetal linkages [14]. This crosslinking reaction reduces intermolecular sliding, significantly improving the stability and durability of the aerogel network [15]. For instance, studies have shown that aerogels crosslinked with GA exhibit higher void ratios and enhanced performance compared to non-crosslinked ones [16]. Therefore, improving the mechanical properties and multifunctionality of chitosan-based aerogels through composite formation and crosslinking is critical for their wider application in fresh fruit and vegetable packaging.

Meanwhile, metal nanoparticles have attracted considerable attention for their excellent antibacterial activity, but their high mobility and potential cumulative toxicity limit their safe use in chitosan-based aerogel packaging. Two-dimensional nanomaterials such as montmorillonite (MMT) possess unique layered structures that can physically confine and coordinate metal ions, enabling controlled and sustained release that reduces toxicity and improves safety [17]. Moreover, MMT can serve as a nanofiller in chitosan-based aerogel packaging to enhance mechanical performance and functional properties. For example, Ternary composite aerogels comprising graphene oxide, montmorillonite, and carboxymethyl chitosan were prepared, and MMT incorporation was shown to markedly enhance mechanical strength and adsorption capacity, underscoring its effectiveness as a functional nanofiller in chitosan-based aerogel packaging [17].

**Figure 1:**
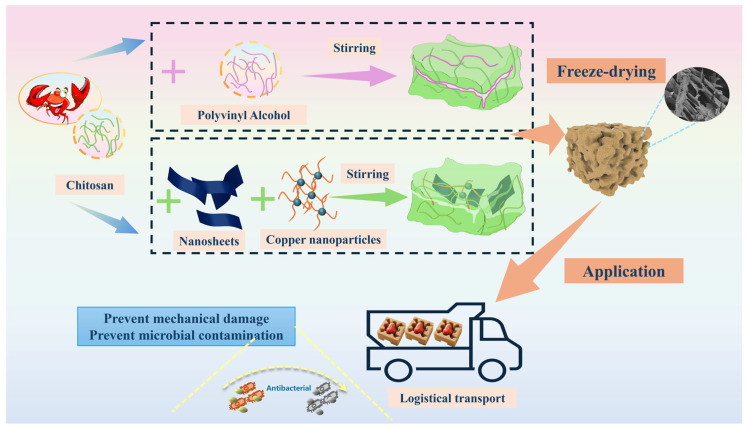
Cushioning chitosan aerogel for postharvest quality maintenance of wax apples.

This study aims to develop biodegradable chitosan-based aerogel materials with cushioning protection and antimicrobial preservation functions to address the challenges of mechanical damage and spoilage of fruit and vegetables during transportation and storage (Figure 1). Chitosan was used as the matrix to develop and optimize two types of functionalized modified aerogel cushioning pads, and their potential applications in cushioning protection and preservation of wax apples were explored.

## 2. Materials and Methods

### 2.1. Materials

The materials used in this experiment were as follows: Chitosan (deacetylation degree ≥ 85%) was purchased from Shandong Haiyihua Biotechnology Co., Ltd. (Jinan, China). Glacial acetic acid was purchased from Guangdong Guanghua Sci-Tech Co., Ltd. (Guangzhou, China). Montmorillonite was purchased from Shanxi Xiya Chemical Co., Ltd. (Xi’an, China). Polyvinyl alcohol (PVA, type 1799, corresponding to an average molecular weight of approximately 74,000–75,000 g/mol) with an alcoholysis degree of 98–99% (mol/mol) was purchased from Shanghai Aladdin Biochemical Technology Co., Ltd. (Shanghai, China). Cetyltrimethylammonium bromide (CTAB) with a molecular weight of 364.45 g/mol was purchased from Shanghai Aladdin Biochemical Technology Co., Ltd. (Shanghai, China). Glutaraldehyde was purchased from Shanghai Jizhi Biochemical Technology Co., Ltd. (Shanghai, China). TEMPO-oxidized nanocellulose and copper nanoparticles were provided by Nanjing Agricultural University (Nanjing, China). All other chemicals were of analytical grade.

### 2.2. Preparation of Chitosan-Based Aerogels Buffer Packaging

#### 2.2.1. Preparation of Chitosan-Polyvinyl Alcohol Aerogel

The preparation of chitosan-polyvinyl alcohol aerogels was carried out according to the method with slight modifications [18]. Briefly, CS solutions (1, 2, and 4 wt%) were first prepared by dissolving CS powder in a glacial acetic acid aqueous solution (1.0% *v*/*v*) under magnetic stirring at room temperature for 4 h. PVA solutions (1, 2, and 4 wt%) were separately prepared by dispersing PVA powder in deionized water, followed by heating at 90 °C under continuous stirring for 2 h until clear. Subsequently, equal volumes of CS and PVA solutions at the same concentration were mixed thoroughly. The freeze-drying process comprised an initial pre-freezing step at −80 °C for 12 h, followed by primary drying where the sample temperature was maintained at −50 °C under a chamber vacuum of 10 Pa for 48 h, to obtain aerogels with various compositions. This procedure yielded CP aerogels with final solid contents of 1%, 2%, and 4%. Based on this, the solid content was fixed at 2% CS and PVA solutions with 2% solid content were then mixed at volume ratios of 2:1, 1:1, and 1:2, respectively, followed by freeze-drying to obtain CP aerogels with different mixing ratios, completing the screening of the chitosan to PVA ratio. Finally, under the conditions of 2% solid content and a 1:1 volume ratio, GA was added as a cross-linking agent at 1%, 3%, and 6% of the solid content. The cross-linking reaction was allowed to proceed at room temperature under continuous stirring for 6 h, with the pH of the mixture naturally maintained at approximately 4–5 due to the acetic acid solvent. After the reaction, the mixtures were freeze-dried to obtain CP aerogels with different GA contents (1% GA, 3% GA, and 6% GA CP aerogels), completing the optimization of the cross-linking agent amount.

#### 2.2.2. Preparation of Chitosan-Montmorillonite/Copper Nanoparticles Aerogel

Montmorillonite/copper nanoparticle aerogels were prepared according to a reported method [17], with slight modifications. First, a 2 wt% CS solution was prepared as described in Section 2.2.1. Prior to composite formation, MMT was organically modified with cetyltrimethylammonium bromide (CTAB). In a typical modification, 0.5 g of MMT was dispersed in ultrapure water, followed by the addition of 0.075 g of CTAB. The mixture was stirred at 60 °C for 3 h. The resulting modified MMT was collected by filtration, washed thoroughly with ultrapure water, and stored at 4 °C. This modified MMT was then used as the additive. Different amounts of modified MMT (1%, 5%, 10%, and 20% of the CS solid content) were used. Subsequently, CuNPs-TNF solutions were added at proportions of 1%, 5%, 10%, and 15% (based on CS solid content) and stirred thoroughly. The mixtures were poured into a 24-well plate and then subjected to freeze-drying. The process included an initial pre-freezing step at −80 °C for 12 h, followed by primary drying at −50 °C under a vacuum of 10 Pa for 48 h, to obtain aerogels with various compositions.

### 2.3. Characterization of Aerogels

All characterization tests were performed on the freeze-dried aerogels. The cylindrical hydrogel precursors formed in a 24-well plate were converted into aerogels by lyophilization. To preserve their dry state and prevent moisture absorption, the entire plate containing the aerogels was immediately sealed with parafilm and stored in a desiccator until the moment of testing.

#### 2.3.1. Scanning Electron Microscope

The microscopic pore structure of the aerogels was observed using a scanning electron microscope (SEM, Hitachi Regulus 8100, Tokyo, Japan). For the observations, the surfaces of the samples were directly used, while cross-sections were obtained by cutting the aerogels. Both the surface and cross-sectional samples were then fixed to the sample holder using conductive adhesive and sputter-coated with gold [19]. The morphologies of the samples were imaged under various magnifications.

#### 2.3.2. Density

The dimensions of the aerogels were measured using a vernier caliper, and their masses were determined using an analytical balance (accuracy: 0.0001 g) [20]. The density ( ρ) was calculated using the formula

$$\rho =\frac{m}{v}$$

where *ρ* is the aerogel density (g/cm^3^), *m* is the mass (g), and *v* is the volume (cm^3^).

#### 2.3.3. Porosity

The initial weight of the aerogel (*m*_1_) was recorded, and the sample was immersed in absolute ethanol until fully saturated. It was then weighed again (*m*_0_) [21]. The porosity was calculated using the formula

$$P(\%)=\frac{m_{0}-m_{1}}{\rho v}\times 100$$

where *ρ* is the density of absolute ethanol (0.7893 g/cm^3^) and *v* is the volume of the aerogel (cm^3^).

#### 2.3.4. Mechanical Properties

A texture analyzer (TMS-PRO, FTC, Sterling, TX, USA) was employed to evaluate the mechanical properties via a cyclic extrusion test. The test was performed with a 50 N load cell. The specific settings were as follows: a pre-test speed of 1 mm/min, a test speed of 10 mm/min, a return speed of 10 mm/min, an initial trigger force of 0.5 N, and a compression strain of 30% (based on the initial sample height). The compression direction was perpendicular to the aerogel plane. Each sample underwent 5 consecutive compression cycles to assess its compression-recovery behavior [22].

### 2.4. Wax Apple Preservation Experiment

#### 2.4.1. Simulated Transportation Test

Wax apple fruits (*Syzygium samarangense* Merr. & L.M.Perry cv. ‘Mifengling’) at commercial maturity in July 2024 were harvested from an orchard in Dongshan County, Fujian, China, and transported to the laboratory within 24 h. Fruits of uniform size and maturity, free from defects, were selected. The selected fruits had an average weight of 80.57 ± 5.27 g, firmness of 21.38 ± 0.64 N, and total soluble solids content of 9.37 ± 0.76 °Brix. They were then randomly divided into five groups. A control group was prepared without any cushioning material, while for the remaining four treatments, each fruit was individually placed in a plastic container lined with a specific aerogel cushion: pure chitosan, chitosan-polyvinyl alcohol, or chitosan-montmorillonite/copper nanoparticle aerogel. All containers were then subjected to simulated road transport on a versatile orbital shaker (Model HY-3, Changzhou Guohua, Changzhou, China) at 210 rpm for 1 h and subsequently stored at 20 ± 0.5 °C and 85 ± 2% relative humidity. Samples were collected on 0 d, 2 d, 4 d, 6 d, and 8 d for visual and quality assessments.

#### 2.4.2. Decay

Decay incidence was assessed at each sampling day (0, 2, 4, 6, and 8 d of storage). Fruits exhibiting symptoms such as water soaking, visible decay, or mold growth were classified as rotten [23]. The decay (%) was calculated using the following formula:

$$\mathrm{Decay}\;(\%)=\frac{Rotten\;fruit\;quantity}{Total\;fruit\;quantity}\times 100$$

#### 2.4.3. Weight Loss

Weight loss was determined by direct weighing using an electronic balance (LE3002E, Mettler-Toledo, Greifensee, Switzerland; precision: 0.01 g) [24]. It was calculated using the following formula:

$$Weight\;loss\left(\%\right)=\frac{W_{0}-W_{t}}{W_{0}}$$

where W_0_ is the initial quality of fruit, g; W_t_ is the quality of fruit for storage days

#### 2.4.4. Firmness

Fruit firmness was measured using a Brookfield CT3 texture analyzer (Ametek Brookfield Instrument, Middleboro, MA, USA). The texture analyzer was equipped with a 5 mm cylindrical probe. The testing conditions included a trigger force of 0.5 N, test speed of 1.0 mm/s, and a pressing depth of 5 mm [25]. Three equatorial points were measured for each fruit. The result was expressed as Newton (N).

#### 2.4.5. Juice Yield

A flesh column was extracted using a puncher and sliced into uniform disks. Six slices were placed into a centrifuge tube pre-filled with absorbent paper and pre-weighed (W_1_). The tube was reweighed (W_2_), centrifuged at 225× *g* for 10 min, and reweighed again after removing the slices (W_3_) [26]. Juice yield was calculated as

$$\mathrm{Juice}\;\mathrm{yield}\;\left(\%\right)=\frac{W_{3}-W_{1}}{W_{2}-W_{1}}\times 100$$

#### 2.4.6. Relative Electrical Conductivity

A slightly modified version of the method was used to determine the relative electrical conductivity of wax apple [27]. Wax apples were cut into 1 mm slices, and 2.0 g of slices were immersed in 20 mL of ultrapure water for 2 h. The conductivity was measured and recorded as P_1_. The solution was then boiled for 10 min, cooled for 15 min, and measured again as P_2_. The relative conductivity was calculated as

$$P=\frac{P_{1}-P_{0}}{P_{2}-P_{0}}\times 100\%$$

where P_0_ is the conductivity of ultrapure water (μS/cm).

#### 2.4.7. Surface Lightness

A handheld colorimeter (model CR-400; Konica Minolta, Tokyo, Japan) was used to measure the surface lightness of wax apples. The results were presented in L^∗^ value (lightness) according to the CIE-Lab color scale [28].

#### 2.4.8. Total Soluble Solids (TSS)

A digital refractometer (PAL-RI, Atago Co., Ltd., Tokyo, Japan) was used to measure the total soluble solids content, and the results are reported in °Brix [29]. Juice was extracted by pressing two unspoiled wax apples, and the average of three measurements was recorded.

### 2.5. Statistical Analysis

All experiments were conducted in triplicate, and results were reported as mean ± standard deviation. Statistical analysis was performed using analysis of variance (ANOVA) in SPSS (version 27, IBM Corp., Armonk, NY, USA). Duncan’s multiple range test was used for post hoc comparisons at a significance level of *p* < 0.05. Data visualization was carried out using Origin 2024. In the graphs, significant differences were denoted by different lowercase letters, with identical letters indicating no significant difference.

## 3. Results & Discussion

### 3.1. Characterization of Chitosan and Polyvinyl Aerogel

#### 3.1.1. Optimization of Chitosan and Polyvinyl Alcohol Solid Content

**Figure 2:**
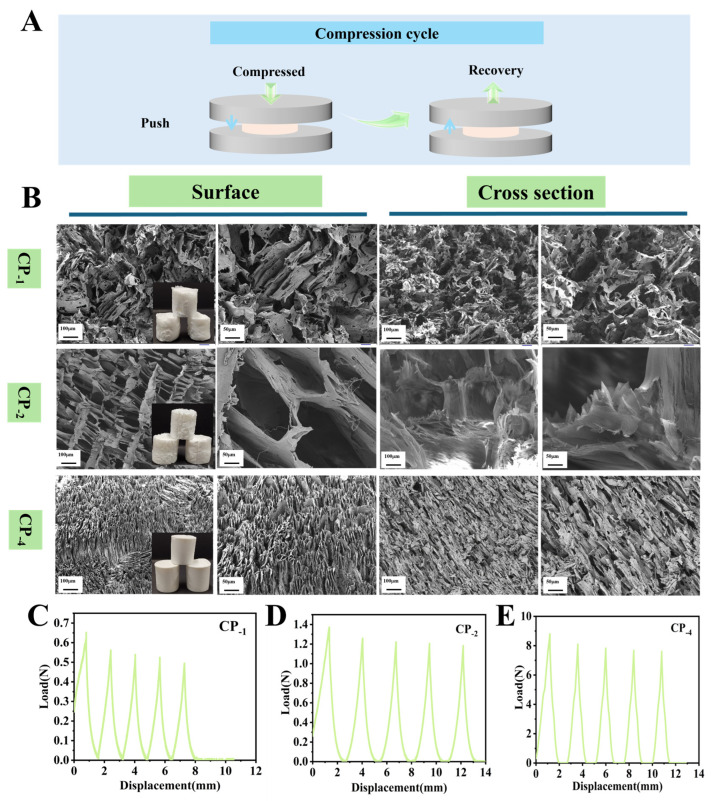
Schematic diagram of aerogel compression process (**A**); Surface morphology and cross section of chitosan-based aerogels with varying chitosan/polyvinyl alcohol aerogel with 1%, 2% and 4% mass fraction (**B**); Cyclic extrusion test results (**C**–**E**).

In the packaging process of fragile fruit, cushioning materials need to ensure softness and at the same time have good structural stability to effectively absorb external forces during transportation or handling and prevent mechanical damage to the fruit [30]. Aerogels have attracted much attention because of their ultra-light weight, adjustable structure and excellent cushioning properties, but their mechanical properties depend largely on the microstructure, especially the pore characteristics and network solidity [31]. Materials with excessive stiffness may crush the fruit, while insufficient stiffness lacks the necessary support. Therefore, optimizing the pore structure and mechanical properties of aerogels is the key to enhance their cushioning performance. The microstructures of chitosan and polyvinyl (CP) aerogels (CP-1, CP-2, CP-4) prepared with different solid contents (1%, 2%, and 4%) were analyzed, as shown in Figure 2B. The CP-1 sample exhibited an extremely loose structure with a poorly defined porous network. This was likely due to the low solid content of the CS/PVA blend, which increased the distance between molecular chains and reduced the number of crosslinking points, thereby forming a sparse three-dimensional network insufficient to support structural integrity. In contrast, the CP-2 aerogel displayed a more uniform and well-developed porous structure. At this moderate polymer concentration, chitosan and PVA chains were more likely to form a continuous network via hydrogen bonding and electrostatic interactions, resulting in an appropriate crosslinking density and improved structural stability [32]. Moreover, the solution concentration was adequate to support uniform nucleation, promoting the formation of a homogenous pore structure as ice crystals sublimated during the freeze-drying process. However, CP-4 presented an excessively dense structure with minimal visible pores. This was attributed to the high viscosity of the precursor solution, which restricted the mobility of polymer chains and led to excessive local crosslinking and aggregation, rather than the formation of a uniform porous network [33]. Additionally, the high concentration accelerated the gelation process, hindering solvent escape and resulting in a closed-pore morphology.

The mechanical strength and compressive resilience of the aerogels were evaluated through cyclic compression tests (Figure 2C–E). The CP-1 sample showed a low maximum compressive force of only 0.7 N and exhibited poor cyclic performance, consistent with its highly porous and unstable structure. In comparison, CP-2 and CP-4 exhibited higher maximum compressive forces of 1.4 N and 8.8 N, respectively. Although CP-4 had the highest compressive force, it demonstrated inferior cyclic stability compared to CP-2, making it less suitable for cushioning applications that require repeated deformation resistance. Based on a comprehensive evaluation of pore morphology, compressive strength, and elastic recovery behavior, CP-2 was identified as the optimal CS-based aerogel formulation for further use in cushioning packaging. These findings suggest that the crosslinking interactions between CS and PVA facilitate the formation of a uniform, compact, and well-defined porous structure, which plays a critical role in enhancing the mechanical performance of the aerogels.

#### 3.1.2. Optimization of Chitosan/Polyvinyl Alcohol Mass Ratio

**Figure 3:**
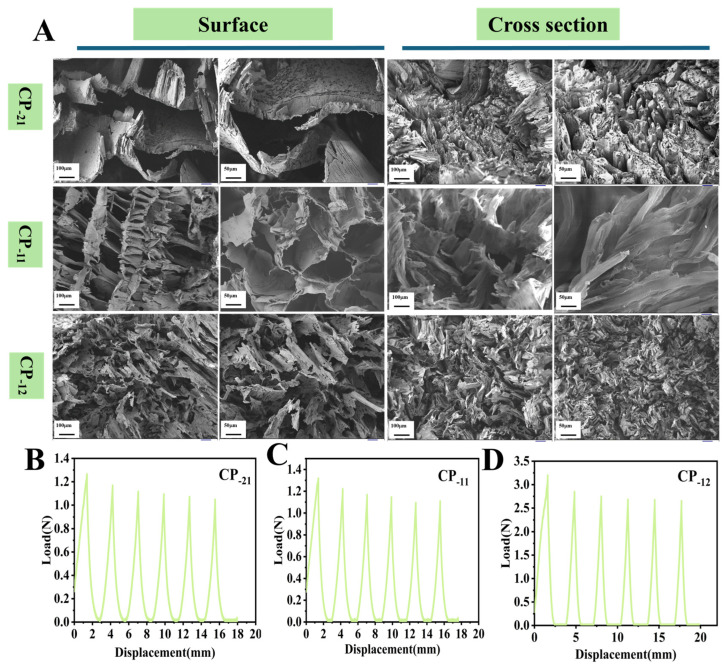
Surface morphology and cross section of chitosan-based aerogels with 2:1, 1:1, 1:2 ratio of chitosan and polyvinyl alcohol aerogels (**A**); Cyclic extrusion test results (**B**–**D**).

With the total solid content fixed at 2%, the mass ratios of CS to PVA were adjusted to 2:1 (CP-21), 1:1 (CP-11), and 1:2 (CP-12). As shown in Figure 3A, the CP-2 aerogel exhibited an uneven pore distribution. This was attributed to the high CS content, which resulted in excessive exposure of amino groups and the formation of a compact hydrogen-bonded network with the hydroxyl groups of PVA [30]. The strong intermolecular interactions led to molecular aggregation, which in turn inhibited pore formation during freeze-drying. In contrast, the CP-11 aerogel displayed a relatively uniform and well-developed porous structure. This was likely due to the near 1:1 molar ratio of amino groups in CS and hydroxyl groups in PVA, facilitating the formation of a periodic hydrogen bonding array. The moderate crosslinking density provided by this ratio allowed directional pore growth, guided by ice crystal formation during the freeze-drying process [34]. The CP-12 aerogel, on the other hand, presented a disordered lamellar morphology with many isolated, non-interconnected layers. This structure likely resulted from the excess PVA forming a continuous phase with high crystallinity, which hindered the formation of a coherent CS-based network. Additionally, the low CS content provided insufficient crosslinking points and weakened chain entanglement, further compromising the structural integrity of the aerogel. The mechanical properties of the aerogels were evaluated through cyclic compression testing (Figure 3B–D). The maximum compressive forces of CP-21, CP-11, and CP-12 were 1.3 N, 1.3 N, and 3.2 N, respectively. Although CP-12 exhibited the highest compressive strength, CP-11 demonstrated a more favorable balance between structural uniformity and mechanical resilience. Therefore, CP-11, with a CS: PVA mass ratio of 1:1, was identified as the optimal formulation and was selected for subsequent experiments.

#### 3.1.3. Optimization of Crosslinker Content

**Figure 4:**
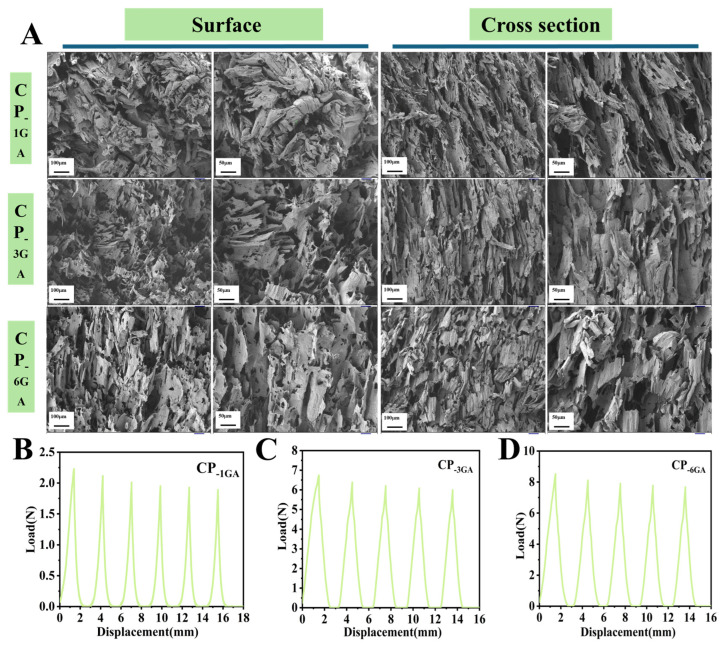
Surface morphology and cross section of varying with glutaraldehyde content of 1%, 3% and 6% of chitosan and polyvinyl alcohol aerogels (**A**); Cyclic extrusion test results (**B**–**D**).

The effect of GA content on the microstructure and mechanical properties of CP aerogels was investigated. Aerogels with different GA concentrations (1%, 3%, and 6%) were prepared and designated as CP-1GA, CP-3GA, and CP-6GA, respectively. As shown in Figure 4A, all three aerogels exhibited similar lamellar microstructures, indicating that GA crosslinking did not markedly affect the overall pore morphology. To further assess their mechanical performance, cyclic compression tests were conducted (Figure 4B–D). The maximum compressive forces of CP-1GA, CP-3GA, and CP-6GA were 2.2 N, 6.7 N, and 8.5 N, respectively, representing approximately 2-, 5-, and 6-fold increases compared to the uncross linked CP-11 aerogel (1.3 N). Both 3% and 6% GA significantly improved the mechanical strength and cyclic stability of the aerogels, suggesting that GA served as an effective chemical crosslinker for enhancing network density and mechanical robustness. However, considering the need to minimize the amount of chemical crosslinker while maintaining adequate performance, the 3% GA formulation was selected for subsequent applications.

In summary, the optimized CP aerogel formulation—with 2% total solid content, a 1:1 mass ratio of CS to PVA, and 3% GA as the crosslinking agent—resulted in an aerogel that is structurally stable and mechanically resilient. This aerogel features a uniform porous structure and a compressive strength of 6.7 N, making it highly suitable for cushioning applications in the packaging of delicate produce such as fruit and vegetables.

### 3.2. Characterization of Chitosan-Montmorillonite/Copper Nanoparticles Aerogel

#### 3.2.1. Optimization of Montmorillonite Content

**Figure 5:**
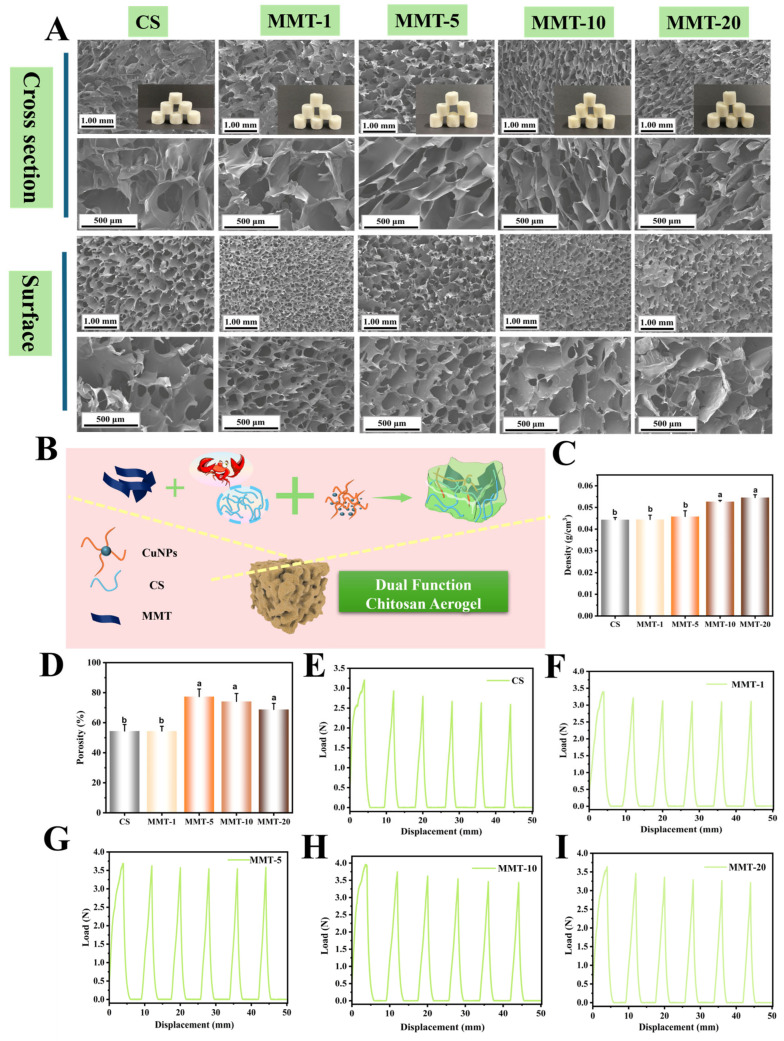
Cross-sectional and surface morphology of pure chitosan aerogel and chitosan-based aerogels with varying MMT additions: MMT-1, MMT-5, MMT-10, and MMT-20 (**A**); Schematic illustration of the preparation of MMT/CuNPs-TNF aerogels (**B**); Density (**C**), porosity (**D**), and cyclic extrusion test results of pure chitosan aerogel and MMT-reinforced aerogels (**E**–**I**). The mean ± standard deviation (*n* = 3), different lowercase letters indicate significant difference (*p* < 0.05).

MMT is a two-dimensional layered nanoclay whose unique lamellar structure can enhance the structural integrity of aerogels [35]. Additionally, CS and MMT can form intermolecular cross-links through hydrogen bonding and electrostatic interactions, further strengthening the three-dimensional network of the aerogel. SEM imaging (Figure 5A) was used to investigate the structural characteristics of CS-based aerogels containing different MMT concentrations (0%, 1%, 5%, 10%, and 20%). The pure CS aerogel displayed a locally unidirectional porous structure with large, loosely arranged pores. Upon MMT incorporation, the internal structure became more uniform and compact, characterized by a honeycomb-like morphology. This improvement was attributed to the two-dimensional, flake-like nature of MMT, whose layered structure and interlayer electrostatic forces provided internal support, thereby improving the rigidity and structural stability of the aerogel. Among the tested formulations, the aerogel with 5% MMT showed the most desirable structural features, exhibiting a densely packed and well-organized tubular morphology in cross-section, along with relatively uniform surface pores. However, when the MMT content increased to 20%, partial collapse of the porous network was observed, accompanied by larger and irregularly shaped pores. This degradation was likely due to poor dispersion of the excessive MMT, which disrupted the interaction between the 2D MMT nanosheets and the 1D CS molecular chains, ultimately hindering the formation of a homogeneous and stable pore network. The influence of MMT on aerogel density was presented in Figure 5C. All aerogels exhibited low densities ranging from 0.04 to 0.06 g/cm^3^. As the MMT content increased, the density of the aerogels gradually rose, reaching 0.06 g/cm^3^ at 20% MMT, likely due to increased solid content and improved structural compactness. Figure 5D showed the porosity values of the pure CS aerogel and the MMT-reinforced samples. The porosities of CS, MMT-1, MMT-5, MMT-10, and MMT-20 were 54.4%, 54.4%, 77.4%, 74.2%, and 68.8%, respectively. Compared to the pure CS aerogel, all MMT-added samples showed increased porosity. This was attributed to the line-surface interweaving between MMT sheets and CS chains, which enriched and refined the pore structure. Among them, the MMT-5 aerogel exhibited the highest porosity, which aligned well with the SEM results. As the MMT content continued to increase, porosity decreased, and the internal structure became rougher, possibly due to MMT agglomeration. Previous studies reported that high porosity was beneficial for improving the mechanical resilience of aerogels [36]. To evaluate mechanical performance, five-cycle compression tests with 30% deformation were conducted using a texture analyzer (Figure 5E–I). This deformation range was selected to simulate the mechanical stress typically encountered by cushioning materials during fruit and vegetable transportation [31]. The results demonstrated that the pure CS aerogel exhibited poor cyclic stability and recovery, while the addition of MMT significantly enhanced its mechanical performance. These findings suggested that the MMT-5 aerogel had superior cushioning capacity, making it a promising candidate for protecting the fragile tissues of wax apples during postharvest handling and transportation.

In summary, although CP exhibited the highest compressive strength (Figure 4C), its cyclic stability was inferior to that of MMT-5. MMT-5 also demonstrated superior elasticity and recoverability. Therefore, copper nanoparticles with known antimicrobial properties were incorporated into the MMT-5 matrix to develop a composite material that offers both cushioning and antimicrobial functions, aiming to protect fragile fruit that are susceptible to mechanical damage and microbial contamination.

#### 3.2.2. Optimization of Copper Nanoparticles Antibacterial Fiber Content

**Figure 6:**
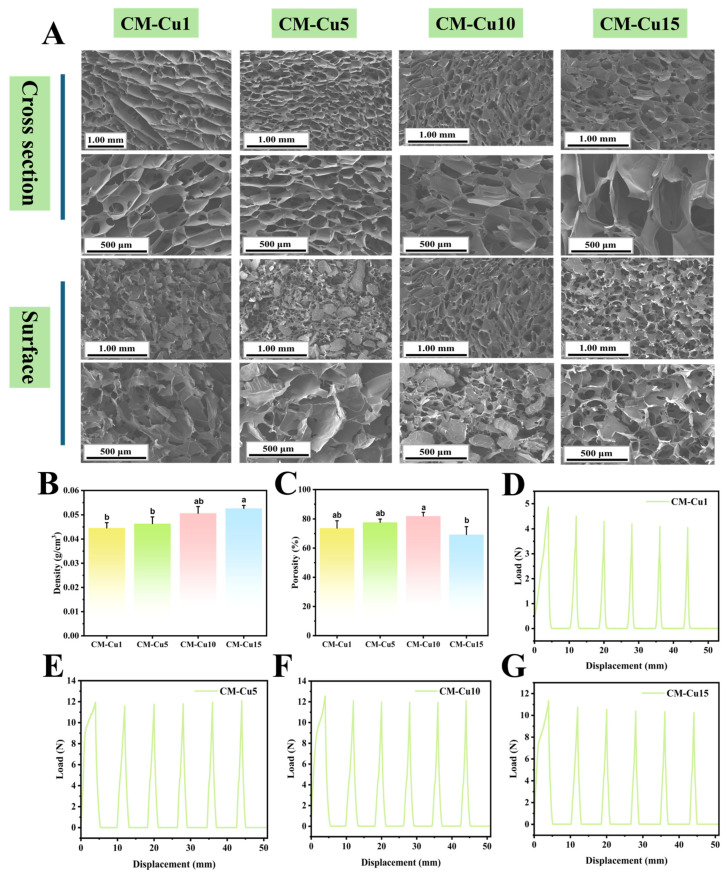
Cross-sectional and surface morphology of pure chitosan aerogel and chitosan-based aerogels with varying CuNPs-TNF additions: CM-Cu1, CM-Cu5, CM-Cu10, and CM-Cu15 (**A**); Density (**B**), porosity (**C**), and cyclic extrusion test results (**D**–**G**). The mean ± standard deviation (*n* = 3), different lowercase letters indicate significant difference (*p* < 0.05).

Fragile fruits are susceptible to mechanical damage and microbial infections and therefore require protective materials with both cushioning and antimicrobial properties. Based on the optimal MMT-5 formulation, the effects of different addition levels (1%, 5%, 10%, and 15%) of CuNPs-TNF antibacterial fibers on the morphology and pore structure of chitosan-montmorillonite/copper nanoparticles aerogels (CM-Cu) were investigated. As shown in Figure 6A, the CM-Cu5 aerogel exhibited a neatly arranged cross-sectional pore structure with uniform pore sizes and a relatively three-dimensional framework; however, its surface morphology was less ideal. In contrast, the CM-Cu10 aerogel displayed a more uniformly distributed and interconnected surface porous structure. At the same time, intertwined cellulose nanofibers were clearly visible within the aerogel matrix, contributing to the formation of finer pores and enhanced mechanical integrity. When the CuNPs-TNF content was further increased to 15%, local pore wall collapse and uneven pore distribution were observed. This structural deterioration was likely due to the aggregation of excessive CuNPs-TNF, which disrupted the crosslinking interactions among chitosan, MMT, and nanocellulose, thereby impairing the stability of the aerogel’s three-dimensional network. As shown in Figure 6B, the densities of all CM-Cu aerogel samples remained in the low range of 0.04–0.06 g/cm^3^. With increasing antibacterial fiber content, the density of the aerogels gradually increased. This low-density characteristic is favorable for practical applications, particularly in packaging. Furthermore, the combination of low density and high porosity is advantageous for cushioning performance, as it allows the aerogels to effectively dissipate external forces, making them well-suited for the protection of delicate, perishable products such as fruit and vegetables.

The porosity trends are illustrated in Figure 6C. As the CuNPs-TNF content increased, the porosity of the CM-Cu aerogels first rose and then declined. Notably, the CM-Cu10 sample exhibited the highest porosity, which was beneficial for stress distribution and buffering. In contrast, the porosity of CM-Cu15 decreased, possibly due to the structural interference caused by excessive antibacterial fiber loading, which compromised the aerogel’s pore architecture.

The mechanical properties of the aerogels were assessed through cyclic compression testing, and the results are presented in Figure 6D–G. At 1% CuNPs-TNF addition (Figure 6D), the aerogel showed poor mechanical performance, with a noticeable decline in the compression curve over repeated cycles. When the TNF-CuNP content was increased to 5% and 10% (Figure 6E,F), the curves remained relatively stable, and the CM-Cu10 sample outperformed the CM-Cu5 group in terms of cycle stability and resistance to deformation. This mechanical improvement aligned well with the microstructural observations in Figure 6A. The enhanced performance was attributed to the formation of a finer pore structure by the nanocellulose, which intertwined with chitosan and MMT to reinforce the aerogel matrix [37]. Additionally, nanocopper may have partially filled the nanopores, further strengthening the overall framework. However, when the addition amount reached 15%, the mechanical properties declined again (Figure 6G), likely due to excess CuNPs-TNF interfering with internal bonding, leading to structural instability.

### 3.3. Preservation of Wax Apples

Due to their high respiration and metabolic rates, wax apples are highly perishable and challenging to transport and store. During transportation, they must be protected from external factors such as mechanical vibration and microbial contamination to maintain quality and extend shelf life. Microbial infection in wax apples often results in visible deterioration, including loss of luster and firmness, thereby reducing their market appeal. Given that the two CS-based aerogels exhibit excellent vibration damping properties, and that the CM aerogels are additionally integrated with antimicrobial fibers, wax apples were selected as a model fruit to evaluate the practical preservation performance of the different aerogels. Specifically, Control, CS, CP, CM, and CM-Cu aerogels were compared in terms of their effectiveness in protecting and preserving wax apples during storage and transport.

#### 3.3.1. Changes in Appearance and Decay

**Figure 7:**
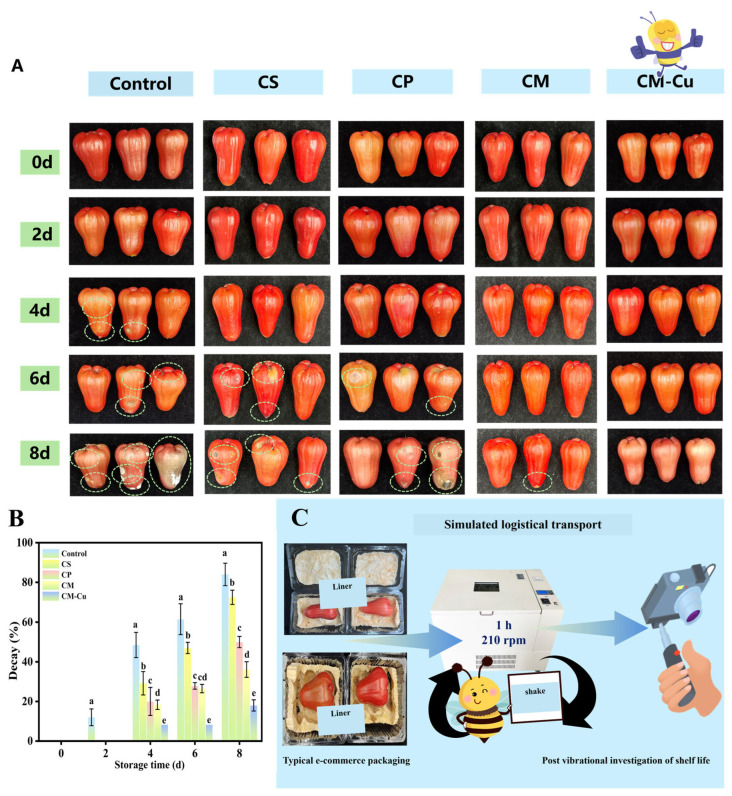
Visual appearance of wax apples stored at 20 ± 0.5 °C treated with storage treated with different aerogels: Control (no aerogel), CS (chitosan aerogel), CP (chitosan-polyvinyl alcohol aerogel), CM (chitosan-montmorillonite aerogel), and CM-Cu (chitosan-montmorillonite/copper nanoparticles aerogel) (**A**); Decay (**B**); Simulated Transportation Test (**C**). The mean ± standard deviation (*n* = 3), different lowercase letters indicate significant difference (*p* < 0.05).

During storage and transportation, wax apples are usually damaged by collisions, extrusions, and impacts, losing the value of the commodity and being more susceptible to microbial infection. Different types of chitosan-based aerogels were investigated for their preservation effects on wax apples as cushioning packaging materials. As shown in Figure 7A, the appearance of wax apples stored for 8 d was recorded. Before and after vibration treatment, wax apples in the aerogel packaging groups maintained good external quality with no visible damage observed. In contrast, the control group exhibited minor surface damage after vibration. During the initial two days, no significant changes in appearance were observed across all treatment groups. On the fourth day, decay symptoms intensified in the control group, primarily concentrated at the apex of the fruit, likely due to greater mechanical impact borne by this more tender region. On the sixth day, white mold spots appeared on wax apples in the CS and CP groups, indicating microbial spoilage. However, the CM and CM-Cu groups showed no visible decay at this time. On the eighth day, the CM-Cu group had a decay of only 18%, whereas the control group exhibited severe decay with a decay of 84% (Figure 7B). These findings demonstrated that the CM-Cu aerogel cushioning packages reduced mechanical damage and exhibited excellent antibacterial properties, effectively prolonging the preservation period of wax apples. The observed suppression of decay in the CM-Cu group can be linked to the incorporated CuNPs. Previous studies have established that CuNPs possess broad-spectrum antibacterial activity [38]. Due to their small size, CuNPs can penetrate bacterial cell walls, leading to wall disruption and membrane damage [39]. Furthermore, interactions between CuNPs and bacterial membranes can reduce transmembrane potential and compromise membrane integrity [40]. Simultaneously, CuNPs release copper ions in solution, which can electrostatically adsorb onto negatively charged bacterial cell membranes, disrupting normal metabolic processes [41]. These ions can also generate reactive oxygen species, resulting in oxidative stress that ultimately kills the bacteria [42]. In summary, chitosan serves as an excellent biomaterial for aerogel preparation, showing great promise in protective food packaging. This study further enhances its functionality by incorporating CuNPs. A synergistic effect is proposed between CuNPs and MMT, where MMT acts as a nanostructured carrier that physically confines and stabilizes CuNPs via interlayer adsorption and surface interactions, thereby modulating their release. Based on these findings, further studies were conducted to evaluate the effects of these aerogels on additional quality parameters during storage.

#### 3.3.2. Fruit Quality Analysis

**Figure 8:**
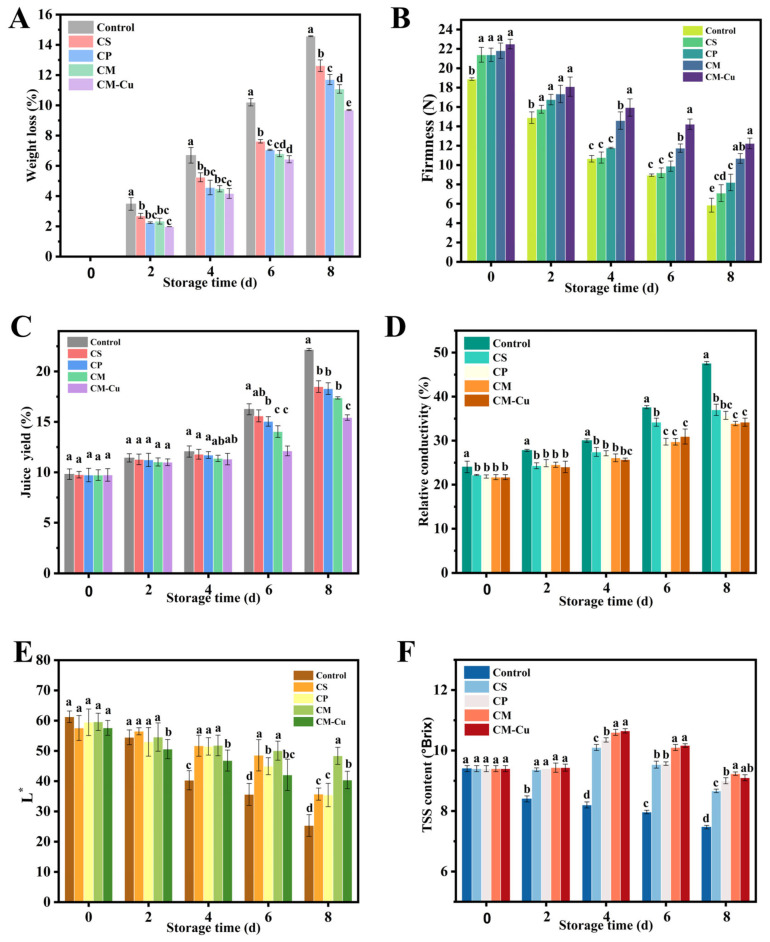
The preservation effect on wax apples stored at 20 ± 0.5 °C treated with Control (no aerogel), CS (chitosan aerogel), CP (chitosan-polyvinyl alcohol aerogel), CM (chitosan-montmorillonite aerogel), and CM-Cu (chitosan-montmorillonite/copper nanoparticles aerogel) (**A**) on wax apples. Weight loss (**A**); Firmness (**B**); Juice yield (**C**); Relative electrical conductivity (**D**); Surface Lightness (**E**); Total soluble solids (**F**). Data are presented as mean ± SE (*n* = 3). Different letters indicate significant differences among groups (*p* < 0.05).

During the storage period, the weight loss of wax apples was closely associated with surface water evaporation and microbial infestation. As shown in Figure 8A, the weight loss in all groups increased progressively over time. The control group exhibited a more rapid weight loss due to surface damage, reaching 15% by the end of the storage period. In contrast, wax apples packaged with aerogel cushioning materials, such as the CP and CM groups, showed less physical damage and lower weight loss of 12% and 11%, respectively, on the eighth day of storage. Notably, the CM-Cu group, which incorporated antibacterial fibers, exhibited the lowest weight loss at 10%, which was lower than that of the other groups, consistent with the decay results. These findings demonstrate that aerogel cushioning packaging effectively delayed moisture loss during storage. This effect is likely attributed to the presence of antimicrobial agents in the CM-Cu aerogel, which suppressed microbial growth, thereby reducing fruit deterioration and associated water loss [34].

Firmness is a critical indicator of wax apple freshness and quality [43]. Throughout storage, firmness decreased in all treatment groups, primarily due to the gradual degradation of cell wall components and reduced intercellular adhesion, leading to fruit softening. On the first day, the control group exhibited lower firmness than the aerogel-treated groups, likely caused by mechanical impact (Figure 8B). On the eighth day, the firmness of the control group declined to 5.7 N, lower than that of all other groups. The marked decrease in the control group’s firmness was attributed to the onset of decay from day 4, during which microbial degradation (e.g., by bacteria and molds) severely damaged the cellular structure and broke down cell wall materials such as pectin, resulting in loosened cell connections and reduced firmness [44].

During storage, the juice yield of wax apples in all groups showed an increasing trend (Figure 8C). By the eighth day, the juice yield of the control group had reached 22%, which was higher than that of the other treatment groups. This was primarily attributed to the earlier occurrence and greater severity of both epidermal damage and microbial decay observed in the control fruits (Figure 7A). The activity of spoilage microorganisms, together with the loss of fruit integrity from mechanical damage, collectively compromised cellular structure and promoted juice leakage, leading to the highest juice yield [45]. In contrast, the aerogel packaging in the CM-Cu group, leveraging its water-absorbing and antimicrobial properties, effectively delayed microbial spoilage and prevented the cross-contamination of juices, thereby better controlling the juice yield [46].

Electrolyte leakage from damaged cell membranes leads to increased electrical conductivity of fruit extracts [47]. Throughout storage, the relative electrical conductivity of wax apples in all groups increased steadily. On the first day, the control group exhibited higher electrical conductivity than the treatment groups, indicating pre-existing membrane damage. As storage progressed, membrane stability declined, causing greater electrolyte leakage and consequent conductivity rise. On the eighth day, the relative electrical conductivity reached 47% in the control group, markedly higher than that in the CP (36%) and CM-Cu (34%) groups (Figure 8D). Figure 8D shows the changes in L* values of wax apples during storage. All groups exhibited a downward trend in L*, corresponding to surface darkening and gloss loss. Color differences were minimal on the first four days. On the eighth day, the control group showed a lower L* than the aerogel-treated groups, suggesting faster pigment degradation due to inadequate protection and tissue damage [48]. The total soluble solids (TSS) of wax apples initially increased and then gradually decreased during storage (Figure 8E), reflecting ripening followed by senescence and continuous respiration consuming TSS. Notably, the CP and CM-Cu groups effectively inhibited the late-stage decline of TSS, thus maintaining the physiological quality of the fruit. These findings are consistent with previous reports on magnetic field effects in strawberries [49] and iceberg lettuce [50]. Compared to CP and CM groups, the CM-Cu aerogel packaging demonstrated superior performance in maintaining wax apple quality by reducing mechanical damage, inhibiting microbial growth, and delaying senescence.

## 4. Conclusions

In this study, two CS-based aerogels were successfully developed and optimized as cushioning and preservation materials for wax apples, a highly perishable tropical fruit. First, the CP aerogel was prepared, with a systematic investigation into the effects of solid content, CS/PVA mass ratio, and crosslinker concentration on the aerogel’s structure and mechanical properties; it exhibited high compressive strength but poor compressive cyclic stability. Interestingly, the introduction of montmorillonite into the chitosan matrix produced a CM aerogel with both high compressive strength and excellent compressive cyclic stability. Finally, copper nanoparticles with known antibacterial properties were added to the CM matrix to create a composite material (CM-Cu) combining cushioning and antimicrobial functions, aimed at protecting delicate fruit vulnerable to mechanical damage and microbial contamination. During storage at 20 °C following simulated transport, the CM-Cu treatments effectively slowed weight loss, maintained fruit firmness, and helped preserve external color and total soluble solids. As a result, the shelf life and overall postharvest quality of wax apples were improved. These findings highlight the potential of chitosan-based aerogel pads as dual-function packaging materials that protect perishable fruit from physical damage and microbial spoilage. It is important to contextualize these results within practical considerations. While the reliance on freeze-drying, though ideal for pore structure formation, is an energy-intensive step that affects the environmental footprint and production scalability. Furthermore, the use of GA as a crosslinker, while efficient, necessitates a thorough safety assessment to meet regulatory standards for food-contact materials. Consequently, future research will focus on exploring alternative processing routes to improve commercial viability, alongside investigating food-grade crosslinkers and expanding the application to other types of perishable produce.

## Data Availability

The original contributions presented in the study are included in the article, further inquiries can be directed to the corresponding author.
